# Impact of an expert-derived, quick hands-on tool on classifying pulmonary hypertension in chest computed tomography: a study on inexperienced readers using RAPID-CT-PH

**DOI:** 10.1007/s11547-024-01852-5

**Published:** 2024-07-24

**Authors:** Lorenzo Cereser, Gaia Zussino, Carmelo Cicciò, Annarita Tullio, Chiara Montanaro, Mauro Driussi, Emma Di Poi, Vincenzo Patruno, Chiara Zuiani, Rossano Girometti

**Affiliations:** 1 Department of Medicine, Institute of Radiology, University of Udine, University Hospital S. Maria della Misericordia, Azienda Sanitaria-Universitaria Friuli Centrale (ASUFC), p.le S. Maria della Misericordia, 15, 33100 Udine, Italy; 2 Department of Diagnostic Imaging and Interventional Radiology, IRCCS Sacro Cuore Don Calabria Hospital, via don A. Sempreboni, 5, 37024 Negrar di Valpolicella, Verona, Italy; 3 Department of Medicine, Institute of Hygiene and Clinical Epidemiology, University of Udine, University Hospital S. Maria della Misericordia, Azienda Sanitaria Universitaria Friuli Centrale (ASUFC), p.le S. Maria della Misericordia, 15, 33100 Udine, Italy; 4 Cardiology, Cardiothoracic Department, University Hospital S. Maria della Misericordia, Azienda Sanitaria Universitaria Friuli Centrale (ASUFC), p.le S. Maria della Misericordia, 15, 33100 Udine, Italy; 5 Department of Medicine, Rheumatology Clinic, University of Udine, University Hospital S. Maria della Misericordia, Azienda Sanitaria Universitaria Friuli Centrale (ASUFC), p.le S. Maria della Misericordia, 15, 33100 Udine, Italy; 6 Pulmonology Department, University Hospital S. Maria della Misericordia, Azienda Sanitaria Universitaria Friuli Centrale (ASUFC), p.le S. Maria della Misericordia, 15, 33100 Udine, Italy

**Keywords:** Pulmonary hypertension, Thoracic imaging, Chest computed tomography, Competency-based medical education

## Abstract

**Purpose:**

To test the inter-reader agreement in classifying pulmonary hypertension (PH) on chest contrast-enhanced computed tomography (CECT) between a consensus of two cardio-pulmonary-devoted radiologists (CRc) and inexperienced readers (radiology residents, RRs) when using a CECT-based quick hands-on tool built upon PH imaging literature, i.e., the “Rapid Access and Practical Information Digest on Computed Tomography for PH-RAPID-CT-PH”.

**Material and methods:**

The observational study retrospectively included 60 PH patients who underwent CECT between 2015 and 2022. Four RRs independently reviewed all CECTs and classified each case into one of the five PH groups per the 2022 ESC/ERS guidelines. While RR3 and RR4 (RAPID-CT-PH group) used RAPID-CT-PH, RR1 and RR2 (control group) did not. RAPID-CT-PH and control groups’ reports were compared with CRc using unweighted Cohen’s Kappa (*k*) statistics. RRs’ report completeness and reporting time were also compared using the Wilcoxon–Mann–Whitney test.

**Results:**

The inter-reader agreement in classifying PH between the RAPID-CT-PH group and CRc was substantial (*k* = 0.75 for RR3 and *k* = 0.65 for RR4); while, it was only moderate for the control group (*k* = 0.57 for RR1 and *k* = 0.49 for RR2). Using RAPID-CT-PH resulted in significantly higher report completeness (all *p* < 0.0001) and significantly lower reporting time (*p* < 0.0001) compared to the control group.

**Conclusion:**

RRs using RAPID-CT-PH showed a substantial agreement with CRc on CECT-based PH classification. RAPID-CT-PH improved report completeness and reduced reporting time. A quick hands-on tool for classifying PH on chest CECT may help inexperienced radiologists effectively contribute to the PH multidisciplinary team.

**Supplementary Information:**

The online version contains supplementary material available at 10.1007/s11547-024-01852-5.

## Introduction

Pulmonary hypertension (PH) is a multifactorial pulmonary vascular disorder that may be idiopathic or related to multiple clinical conditions, represented mainly by cardiac and respiratory diseases [1, 2]. The updated 2022 European Society of Cardiology / European Respiratory Society (ESC/ERS) guidelines define PH as the presence of mean pulmonary arterial pressure (PAP) values greater than 20 mmHg at rest, as determined by right heart catheterization [1]. PH is classified into five groups, including conditions with similar pathophysiological mechanisms and hemodynamic characteristics [1, 3]. The rationale behind this classification scheme relies on providing patients with the most appropriate management and treatment, which largely depend on inherent group-related differences, thus requiring a multidisciplinary approach [1, 3, 4].

When dealing with patients with suspected PH, the diagnostic challenge lies in identifying the causes early to institute prompt treatment [5]. In this light, chest contrast-enhanced computed tomography (CECT) plays a crucial role in suggesting the presence and possible etiology of PH [3, 6, 7] due to its anatomical comprehensiveness, allowing for integrated evaluation of lung, pulmonary vasculature, and mediastinal structures, including the heart [3, 6–8]. Given the clinical relevance of PH, radiologists’ active participation in the multidisciplinary PH team requires adequate knowledge and training in chest CECT imaging.

The appearance of PH on CECT is pleomorphic, encompassing various signs and patterns involving all the thoracic compartments [3, 6–8]. Therefore, one can assume that CECT reporting of PH requires experienced readers. However, the shortage of radiologists dedicated to this topic raises the question of what essential radiological information is needed to enable inexperienced readers to report a PH case knowingly, reliably, and rapidly. It is conceivable that this core consists not just of a set of distinct elements of sophisticated imaging features but of general patterns allowing for relevant clinical information concerning the PH phenotype. To our knowledge, no previous studies assessed the potential benefit inexperienced and/or non-dedicated chest radiologists might have from using an updated hands-on synopsis summarizing and organizing the CECT imaging findings of PH.

The primary aim was to evaluate the impact of using a quick hands-on tool for classifying PH on chest CECT (Rapid Access and Practical Information Digest on Computed Tomography for Pulmonary Hypertension, RAPID-CT-PH) on readers with limited experience in CT imaging and non-dedicated to thoracic imaging. We assessed the inter-reader agreement in assigning the PH group between four radiology residents (RRs) and two radiologists with cardio-pulmonary CT imaging expertise. The secondary objectives were to assess the impact of the RAPID-CT-PH on the agreement between inexperienced and experienced readers in formulating an etiological hypothesis of PH and its effect on the completeness of reports generated by inexperienced readers. Finally, we compared the CECT reading times using or not using the RAPID-CT-PH.

## Material and methods

Our Institutional Review Board approved this monocentric, observational study and waived the acquisition of informed consent from patients due to the retrospective design.

### Study design

**Fig. 1:**
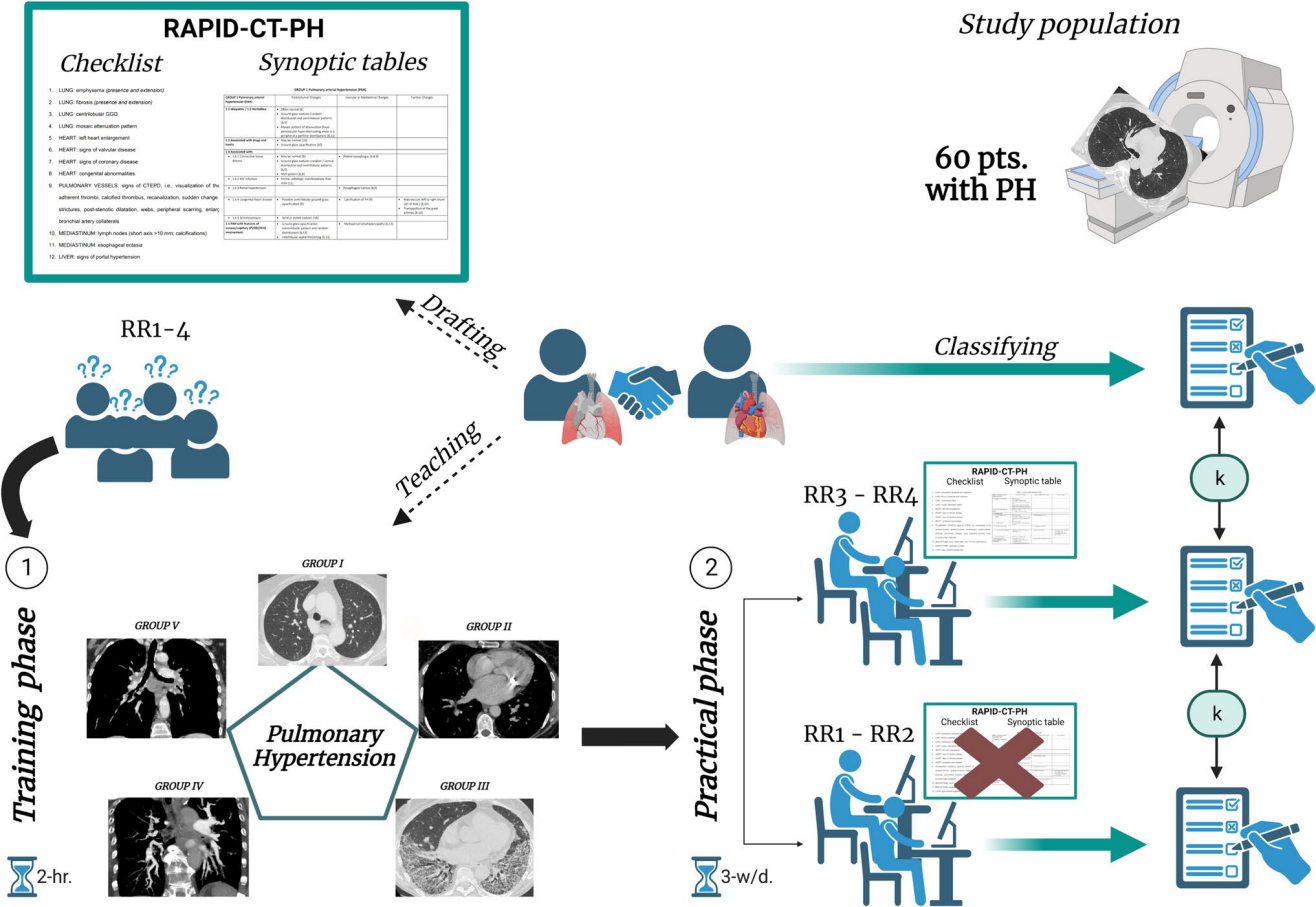
Graphical representation of the study organization (see the main text for details)

Overall, we organized the study as follows (Fig. 1).

**Fig. 2:**
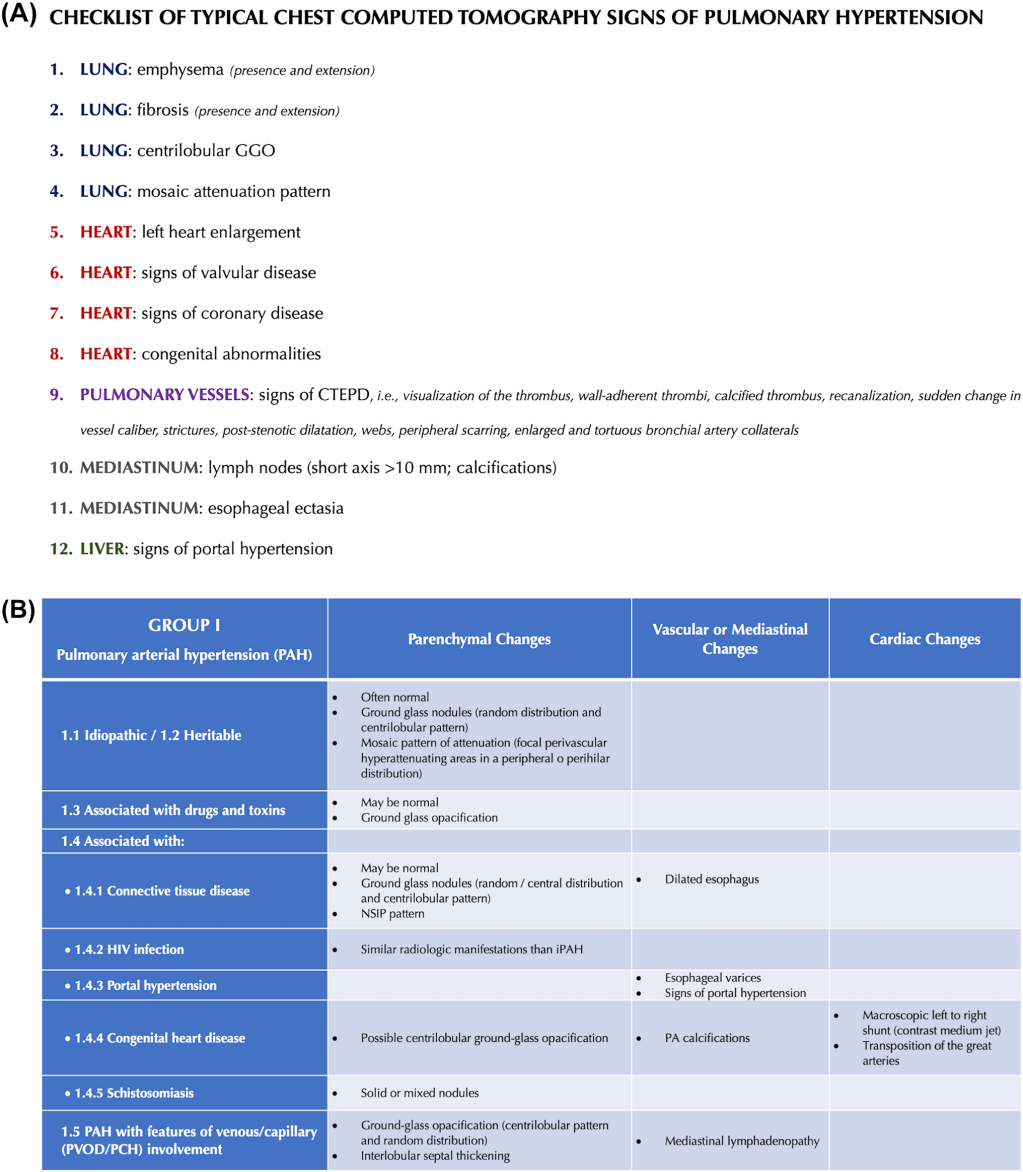

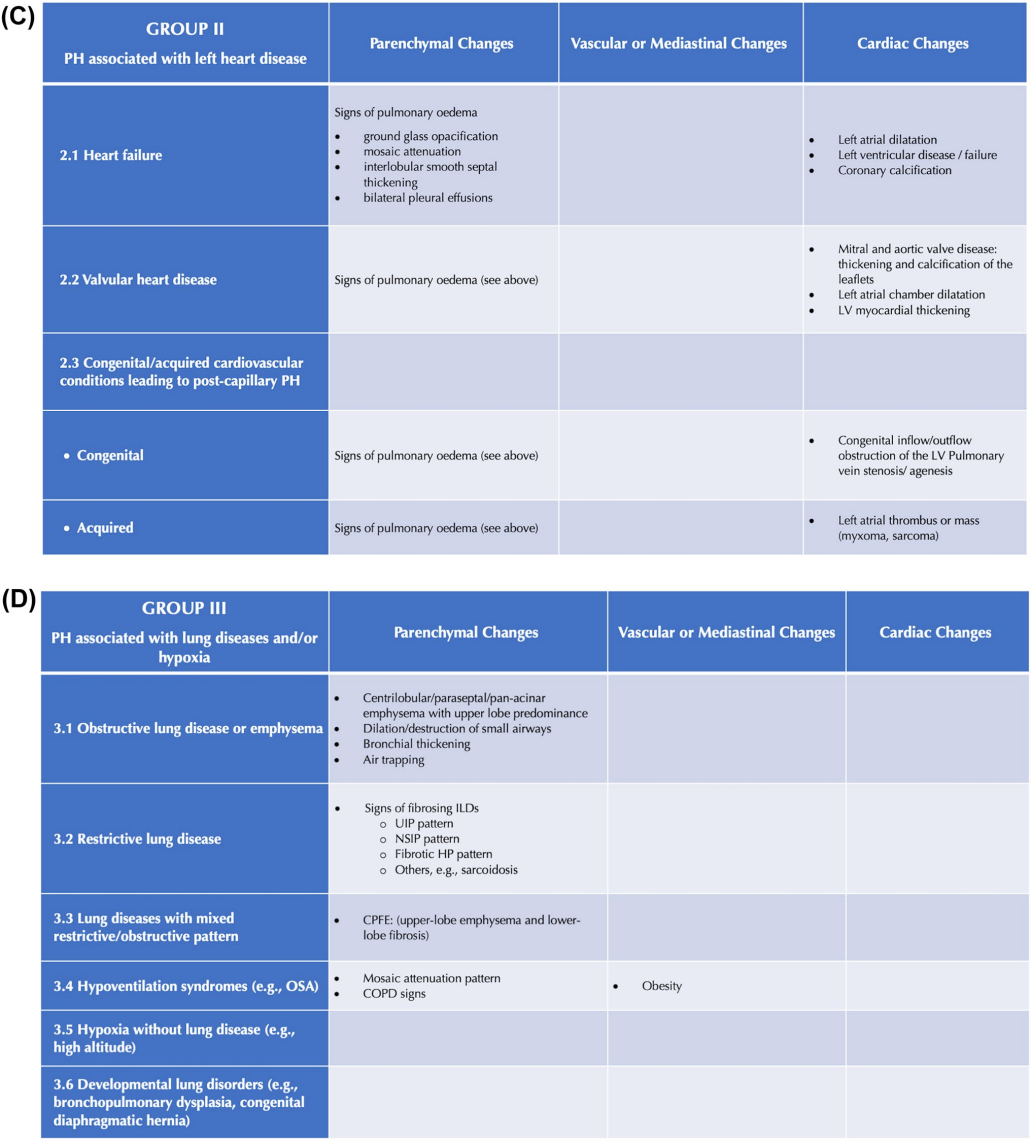

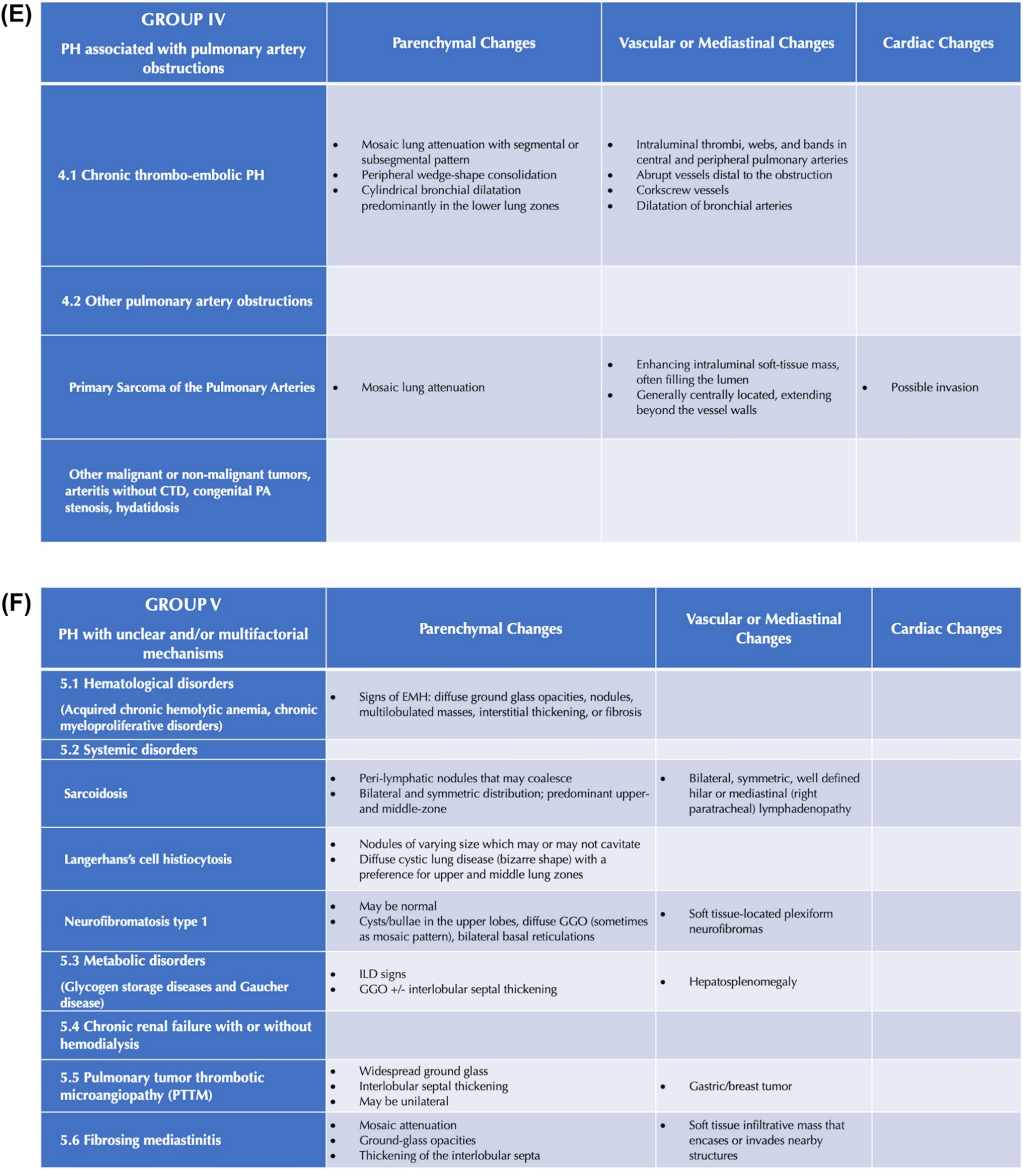
Rapid access and practical information digest on computed tomography for pulmonary hypertension (RAPID-CT-PH). **A** Checklist of typical chest computed tomography signs of pulmonary hypertension. **B**–**F** Synoptic table series categorizing chest computed tomography signs within each PH group and subgroup

First, the study coordinator, i.e., a radiologist not involved in image reading, drafted the RAPID-CT-PH, a PH-dedicated quick hands-on tool based on the current literature evidence [6–25]. Two radiologists with 14 years of experience in cardio-pulmonary CT imaging (chest radiologist 1 [CR1] and chest radiologist 2 [CR2]) actively contributed to this task by reviewing and approving the final draft. The RAPID-CT-PH consisted of two parts: (i) A checklist of typical thoracic signs of PH to be identified on CECT; and (ii) a synoptic table series in which these signs are categorized within each PH group and subgroup. The signs included in the checklist are defined according to the glossary of terms for thoracic imaging from the Fleischner Society [26] and specific papers on PH chest computed tomography findings [6, 8, 12]. The two components forming the RAPID-CT-PH are shown in Fig. 2.

Second, the study coordinator identified four radiology residents (RRs) attending the residency program at our University Hospital, including two senior residents in the last year of the program (radiology resident 1 [RR1] and radiology resident 3 [RR3]) and two novice residents at the beginning of their first year of the program with no previous experience in chest CT imaging (radiology resident 2 [RR2] and radiology resident 4 [RR4]).

Finally, all the residents (RR1-4) participated in a two-phase study, including a dedicated 2-h-long frontal teaching session focused on the chest CECT imaging role in patients with PH (*“training” phase”* of the study) and a subsequent case reading session, with or without the aid of the RAPID-CT-PH (*“practical phase”* of the study). Details are explained below.

### Study population

By performing a computerized search, we identified all consecutive patients aged ≥ 18 years and diagnosed with PH who underwent a chest CECT in our Institution from 2015 to 2022. Patients were referred from the Cardiology Unit or the Rheumatology Clinic of our tertiary care University Hospital. Chest CECT was defined as a CT examination of the whole thorax after intravenous injection of iodinated contrast agent, utilizing two alternative protocols: CT pulmonary angiogram (CTPA) or venous-phase chest CT (VPCT). In the case of multiple available CECT scans, we considered the one closest to the PH diagnosis. According to 2022 ESC/ERS guidelines, PH was defined as the presence of a resting mean PAP > 20 mmHg on right heart catheterization or, in the case of unfeasible catheterization, highly probable PH on echocardiography [1]. Exclusion criteria encompassed unavailability of clinical data, prior cardiac surgery or lung transplantation history, and chest CECT examinations of non-diagnostic quality.

Of the 101 eligible subjects diagnosed with PH, we excluded 17 patients with unavailable CECT, 6 with unavailable clinical data, 3 with acute pulmonary infection potentially masquerading the PH imaging findings, 2 with prior cardiac surgery or lung transplantation history, and 13 because of chest CECT examinations of non-diagnostic quality. Therefore, the final population comprised 60 patients (25 men and 35 women; median age, 70 years; range, 41–85 years). The diagnosis of PH was based on right heart catheterization findings in 39/60 patients (mPAP values: median, 36.8 mmHg; range, 21–68 mmHg), the remaining 21/60 with a high-probability diagnosis on echocardiography. Fifty-three patients underwent chest CECT with CTPA protocol, the remaining 7 being VPCT.

### Chest CECT examination technique

All the chest CECTs were performed on a 64-row computed tomography scanner (Discovery HD750 or Optima, GE Healthcare, Milwaukee, WI, USA), with the patient in the supine position, during inspiratory breath-hold, following intravenous administration of iodinated contrast medium (Iomeprol or Iobitridol, Bracco, Milan, Italy, with an Iodine concentration ranging from 350 to 400 mg/mL). The CTPA and VPCT technical parameters are reported in the supplementary material. The reconstruction sets included images with windowing and kernel dedicated to lung parenchyma (level, − 500 Hounsfield Units [HU]; width, 1700 HU; high-spatial-frequency algorithm) and soft tissues (level, 50 HU; width, 350 HU; standard algorithm). For CTPA examinations, images with arterial pulmonary vessel windowing (level, 100 HU; width, 700 HU) were also obtained.

### Study phases, image analysis, and reference standard

#### Training phase

All the RRs, blinded to the study objectives, attended a dedicated 2 h-long frontal lecture held by CR1 and CR2. The lecture aimed to provide a shared theoretical basis for all the RRs and focused on the role, semiotics, and interpretation of chest CECT in patients with PH.

#### Practical phase

For each reader, the order of case presentation was randomized using freely available software (https://www.randomizer.org).

All RRs, independently and blinded to patient clinical, functional, and imaging data, reviewed all the CECT examinations. Their sole knowledge of clinical data was that all patients had confirmed PH diagnosis. For each CECT examination, the RRs were tasked with formulating a primary hypothesis regarding the PH group assignment. In cases of uncertainty, they were allowed to propose an alternative hypothesis for the PH group. Lastly, the RRs were required to indicate their presumed etiology of PH. For PH case reviewing and reporting, each RR disposed of a days’ number commensurate with the sample size, i.e., three consecutive working days stating 60 cases and a reasonable evaluation of 20 examinations per day.

The RRs were divided into two groups, each consisting of a senior and a novice RR. The groups included the “control” group, composed of RR1 (senior reader) and RR2 (novice reader), without any assistance for reporting the PH cases, and the “RAPID-CT-PH” group, composed of RR3 (senior reader) and RR4 (novice reader), both provided with the RAPID-CT-PH.

The consensus of CR1 and CR2 defined the reference standard (chest radiologists’ consensus, CRc) after they independently reviewed all CECT examinations, blinded to the patient’s clinical history except having been diagnosed with PH. CR1 and CR2 were given a reporting template with designated sections for “description” and “interpretation.” The “description” section comprised the twelve key features constituting the findings’ checklist in the RAPID-CT-PH. In the “interpretation” section, following the same approach as the RRs, they formulated a primary hypothesis regarding the PH group assignment and, in case of uncertainty, could propose an alternative PH group hypothesis. They also indicated the presumed PH etiology. Finally, all discrepancies were resolved through consensus (CRc), and a single PH group allocation hypothesis was selected for each case.

All CECT examinations were evaluated on a Picture Archiving and Communication System workstation (Suitestensa Ebit srl, Esaote Group Company, Genoa, Italy). The same software allowed the readers to multiplanar reconstructions and apply post-processing algorithms such as Maximum Intensity Projection (MIP), Minimum Intensity Projection (MinIP), and Average Intensity Projection (AIP) for more comprehensive image analysis.

### Statistical analysis

The inter-reader agreement between each RR and CRc in classifying PH into the five groups as per the 2022 ESC/ERS guidelines [1] was determined using the unweighted Cohen’s Kappa statistic (*k*) with 95% confidence intervals (CI). Similarly, for the same classification task, we calculated the inter-reader agreement between RR1-RR2-CRc and RR3-RR4-CRc using the unweighted Fleiss’ kappa, and between RRs within the “control” group (RR1-RR2) and “RAPID-CT-PH” group (RR3-RR4) using the unweighted Cohen’s kappa. We deemed the agreement positive when the first or second-choice hypothesis for allocating the PH group provided by the RRs matched that of CRc. The k coefficient was interpreted as < 0.00, poor; 0.00–0.20, slight; 0.21–0.40, fair; 0.41–0.60, moderate; 0.61–0.80, substantial; 0.81–1.00, almost perfect [27].

The inter-reader agreement between each RR and CRc and between RRs within the “control” group and the “RAPID-CT-PH” group in producing an etiological hypothesis of PH was evaluated using Cohen’s kappa statistic. The same methodology outlined in the primary objective subsection was applied to this analysis. We focused on only PH group I and group III cases while excluding PH cases from groups II, IV, and V from the etiology analysis. Indeed, non-cardiac CT suffers from inherent limitations in assessing the heart and coronaries (group II), all the group IV cases in our series were due to CTEPH, and we had only one group V case.

The completeness of reports produced by the RRs was quantified using a completeness index. Initially, the index was expressed on a scale of 0–12 points, with each key feature being assigned one point and later converted to a scale of 0–100. The twelve key features considered were those reported in the RAPID-CT-PH and preliminarily presented to CR1 and CR2 (see above for details). The completeness indexes were calculated for each RR and compared between senior RRs (RR1 vs. RR3) and novice RRs (RR2 vs. RR4) using the Wilcoxon–Mann–Whitney test, as the data distribution did not follow a normal distribution as per the Kolmogorov–Smirnov test.

The average CECT reporting time was compared between RRs with and without the RAPID-CT-PH, i.e., reporting time of RR1-RR2 versus reporting time of RR3-RR4. The Wilcoxon–Mann–Whitney test was used for the comparison due to the non-normal distribution of the data.

To assess consistency between the CRc group hypothesis and the MDT-driven PH final group diagnosis, we calculated the CRc-MDT agreement in grouping PH using the percent agreement (PA) and the Prevalence and Bias Adjusted Kappa (PABAK) with 95%CI. Reference PABAK values were interpreted likewise the k coefficients.

Statistical analyses were conducted using R software version 3.4.2 (R Foundation for Statistical Computing, Vienna, Austria) and Single Case Research—web-based calculators for SCR analysis version 2.0, College Station, Texas. A significance level of 0.05 was used for all tests.

## Results

### CECT-based PH grouping and etiology according to the reference standard

Table 1 presents the distribution of CECT-defined case allocation across the five PH groups based on CRc readings. The two most prevalent PH groups were group II (21/60, 35%), which corresponds to PH associated with left heart disease, and group III (16/60, 26%), representing PH associated with lung diseases and/or hypoxia. Ten out of 60 patients (17%) were categorized as group IV PH, all attributed to chronic thrombo-embolic PH (CTEPH). Only one case was classified as group V PH, resulting from sarcoidosis. The remaining 12 out of 60 (20%) cases fell under group I PH, with connective tissue disease being the most hypothesized etiology (5/12 cases).

**Table 1:** Distribution of chest contrast-enhanced computed tomography-defined case allocation across the five pulmonary hypertension groups based on the chest radiologists’ consensus readings

PH^a^ grouping according to CRc^b^			PH etiology according to CRc	
Group	Definition	N (%)	Subgroup etiology	N (%)
I	Pulmonary arterial hypertension (PAH)	12/60 (20)	1.1 Idiopathic	4/12 (33)
			1.4 Associated with:	
			1.4.1 Connective tissue disease	5/12 (42)
			1.4.3 Portal hypertension	2/12 (17)
			1.4.4 congenital heart disease	1/12 (8)
II	PH associated with left heart disease	21/60 (35)	2.1 Heart failure	14/21 (66)
			2.2 Valvular heart disease	7/21 (33)
III	PH associated with lung diseases and/or hypoxia	16/60 (26)	3.1 Obstructive lung disease or emphysema	4/16 (25)
			3.2 Restrictive lung disease	8/16 (50)
			3.3 Lung diseases with mixed restrictive/obstructive pattern	3/16 (19)
			3.4 Hypoventilation syndromes (e.g., obstructive sleep apnea)	1/16 (6)
IV	PH associated with pulmonary artery obstructions	10/60 (6)	4.1 Chronic thrombo-embolic PH (CTEPH)	10/10 (100)
V	PH with unclear and/or multifactorial mechanisms	1/60 (1)	5.2 Systemic disorders Sarcoidosis	1/1 (100)

^a^Pulmonary hypertension

^b^Chest radiologists’ consensus

The CRc-MDT agreement in grouping PH was almost perfect, with PA = 87% (52/60 cases) and PABAK = 0.83 (95%CI, 0.73–0.93).

### Inter-reader agreement in classifying PH and hypothesizing its etiology

Table 2 reports the distribution of cases across PH groups, as categorized according to RRs. Group 2 PH was the most prevalent, with frequencies ranging from 28 to 50%.

**Table 2:** Distribution of cases across pulmonary hypertension groups, as categorized according to the radiology residents

PH^a^ group	RR^b^ 1 N (%)	RR 2 N (%)	RR 3 N (%)	RR 4 N (%)
I	14/60 (23)	18/60 (30)	16/60 (26)	18/60 (30)
II	23/60 (38)	30/60 (50)	17/60 (28)	17/60 (28)
III	19/60 (15)	8/60 (13)	17/60 (28)	13/60 (21)
IV	12/60 (20)	3/60 (5)	9/60 (15)	11/60 (18)
V	2/60 (3)	1/60 (1)	1/60 (1)	1/60 (1)

^a^Pulmonary hypertension

^b^Radiology resident

Table 3 shows the results of the inter-reader agreement analysis. The inter-reader agreement in classifying PH between the RRs who used RAPID-CT-PH and CRc was substantial for both the novice reader (RR4) and the senior reader (RR3); while, it was only moderate for the readers who did not employ it (RR1 and RR2). When comparing RRs within the same groups, the inter-reader agreement was fair (0.34) in the “control” group (RR1-RR2) and substantial (0.69) in the “RAPID-CT-PH” group (RR3-RR4).

**Table 3:** Results of the inter-reader agreement analysis on pulmonary hypertension group allocation and etiology

	PH^a^ group allocation k [95%CI]	PH etiology k [95%CI]
RR1^b^—CRc^d^ *Cohen’s kappa*	0.57 [0.42–0.73] Moderate	0.43 [0.24–0.62] Moderate
RR2^b^—CRc *Cohen’s kappa*	0.49 [0.33–0.65] Moderate	0.29 [0.12–0.47] Fair
RR1—RR2—CRc *Fleiss’ kappa*	0.46 [0.38–0.55] Moderate	0.31 [0.24–0.38] Fair
RR3^c^—CRc *Cohen’s kappa*	0.75 [0.62–0.88] Substantial	0.74 [0.57–0.91] Substantial
RR4^c^—CRc *Cohen’s kappa*	0.65 [0.50–0.79] Substantial	0.66 [0.48–0.85] Substantial
RR3—RR4—CRc *Fleiss’ kappa*	0.69 [0.61–0.77] Substantial	0.66 [0.57–0.74] Substantial

^a^Pulmonary hypertension

^b^Radiology residents not using RAPID-CT-PH

^c^Radiology residents using RAPID-CT-PH

^d^Chest radiologists’ consensus

The inter-reader agreement with CRc regarding PH etiology was substantial when using RAPID-CT-PH (RR3 and RR4) and only fair-to-moderate for the readers who did not employ it (RR1 and RR2). When comparing RRs within the same groups, the inter-reader agreement was fair (0.25) in the “control” group and moderate (0.58) in the “RAPID-CT-PH” group.

**Fig. 3:**
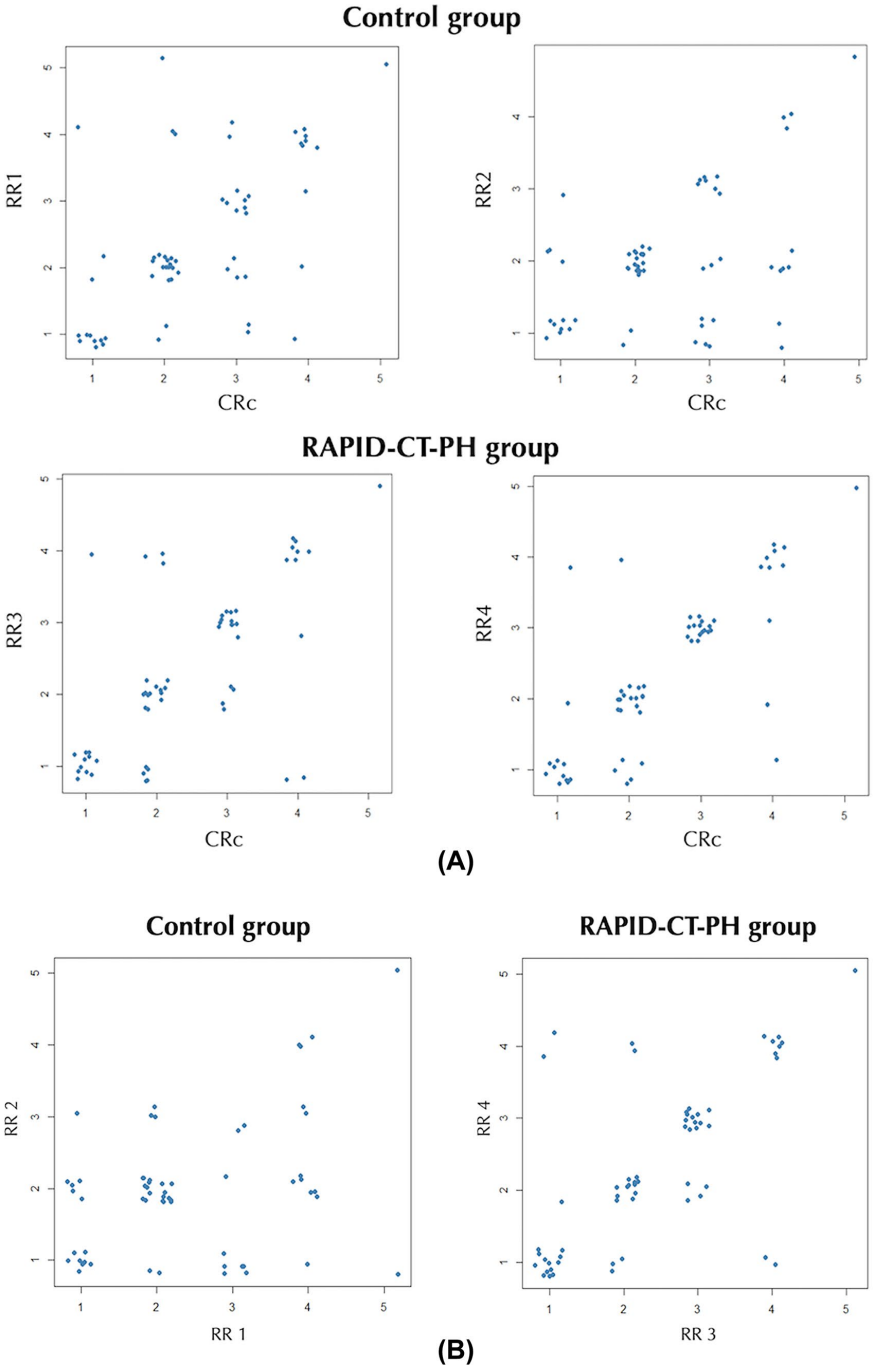
Dispersion diagrams illustrating the differences in agreement across the cases between radiology residents and chest radiologists’ consensus (**A**) and between radiology residents of the same group (control group and RAPID-CT-PH group) (**B**)

**Fig. 4:**
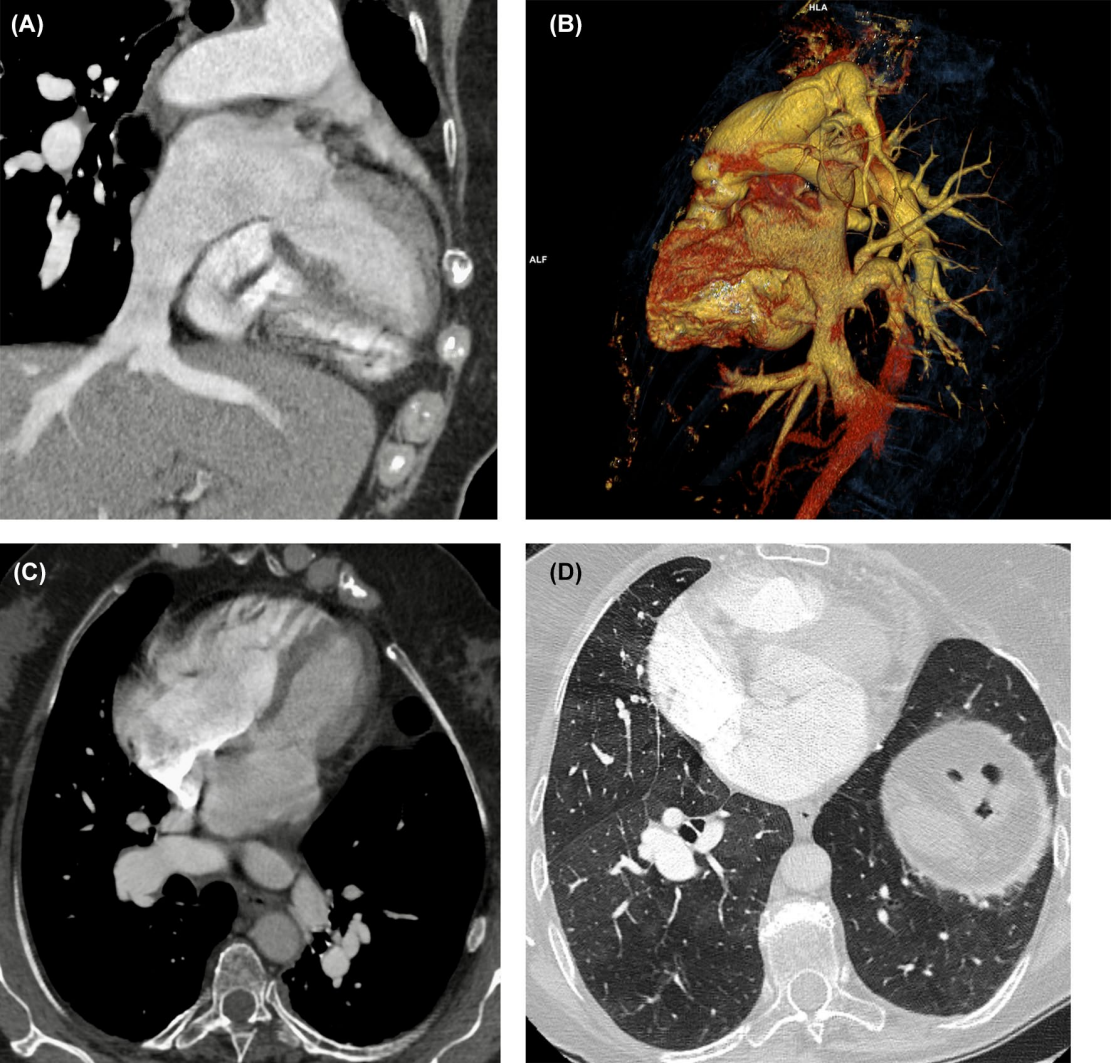
Group I pulmonary hypertension in an 81-year-old woman. Images from CT pulmonary angiography examination show an abnormal return of the inferior vena cava to the left atrium (**A, B**), contrast medium jet directed from the left atrium to the right atrium (**C**), and lung mosaic attenuation pattern (**D**). The radiology residents in the control group did not identify the cardiovascular abnormality (RR2) or misclassified it (RR1); while, the ones in the RAPID-CT-PH group (RR3 and RR4) correctly identified and classified the condition

**Fig. 5:**
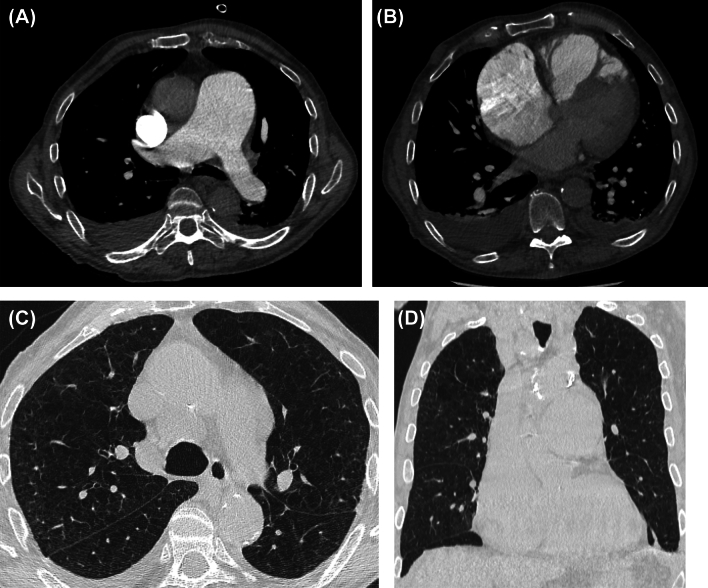
Group III pulmonary hypertension in an 84-year-old man. Images from CT pulmonary angiography examination show enlarged pulmonary artery (**A**), enlarged right heart chambers, with normal appearance of the left ones, and without signs of chronic thromboembolic disease (not shown) (**B**), and diffuse lung emphysema (**C, D**). The radiology residents in the control group (RR1 and RR2) misclassified the heart enlargement as a group II pulmonary hypertension; while, the ones in the RAPID-CT-PH group (RR3 and RR4) correctly identified and classified the condition

Figure 3 visually represents the differences in agreement between RRs and CRc across the cases through dispersion diagrams. Example PH cases are shown in Figs. 4, 5.

### Chest CECT report completeness and reporting time

Table 4 reports the RRs’ report completeness indexes and reporting times. Using RAPID-CT-PH resulted in significantly higher completeness in CECT reports compared to those without it, regardless of RR expertise (all *p* < 0.0001). It also significantly reduced the reporting time (*p* < 0.0001), with a median of 15 min for RRs using RAPID-CT-PH (RR3-RR4) versus 20 min for those who did not (RR1-RR2).

**Table 4:** Radiology residents’ report completeness indexes and reporting times

Report completeness index	Senior residents		Novice residents	
	RR^a^ 1 *without RAPID-CT-PH*	RR 3 *with RAPID-CT-PH*	RR 2 *without RAPID-CT-PH*	RR 4 *with RAPID-CT-PH*
Median (IQR) [min–max]	66.7 (16.7) [33.3–91.7]	100 (0) [100–100]	37.5 (0) [16.7–66.7]	100 (8.33) [83.3–100]
*p*-value^a^	< 0.0001		< 0.0001	

Reporting time *(minutes)*	Residents without RAPID-CT-PH^b^	Residents with RAPID-CT-PH
Median (IQR) [min–max]	20 (5) [10–40]	15 (9.75) [3–30]
*p*-value^*^	< 0.0001	

^a^Radiology resident

^b^Rapid access and practical information digest on computed tomography for pulmonary hypertension

^*^Wilcoxon–Mann–Whitney test

## Discussion

We found that the agreement between readers with limited experience in CT imaging (RRs) and radiologists with expertise in thoracic CT imaging (CRc) regarding chest CECT-based PH classification was substantial when the inexperienced readers utilized RAPID-CT-PH. Conversely, the agreement was only moderate when they did not use RAPID-CT-PH independently from being a novice or senior RR. To our knowledge, no previous studies have investigated the inter-reader agreement for classifying PH on chest CECT nor assessed how a quick hands-on tool could help inexperienced readers with such a task. Our results can be a valuable starting point for developing an educational PH-focused chest CT strategy within radiology residency programs or a practical tool aiding non-chest-devoted radiologists.

Several reasons may explain the higher agreement between RRs and CRc when using RAPID-CT-PH. First, RAPID-CT-PH includes a systematic checklist of CT findings, encompassing the various thoracic anatomical districts. Previous studies on CT imaging of different anatomical regions [28–30] demonstrated that utilizing a checklist of CT findings enables the identification of subtle signs with greater efficiency, going beyond the assessment of apparent findings and, in turn, improving diagnostic quality. Specifically, when dealing with chest imaging, the heart is often under-reported in non-dedicated CT examinations [31]. However, gathering valuable information on cardiac structures is possible even from a “routine” contrast-enhanced chest CT [32–34]. In PH patients, detectable cardiac anomalies include chamber enlargement, coarse valvular or coronary artery calcifications, gross intra- and extracardiac shunts, and atrial thrombi or masses [35]. In addition, explicitly seeking and reporting signs of pulmonary edema may suggest heart failure or valvular heart disease as causes of group II PH [36].

Second, RAPID-CT-PH incorporates a group-based organization of CT findings. This structured layout can assist inexperienced readers in conducting a comprehensive assessment of all the signs related to a particular group, thus integrating information from various anatomical regions. We hypothesize that visually complementing the list of findings in lung parenchyma, vessels, and mediastinum aided the RRs in reliably allocating PH group cases. This could be the case of PH group IV CECTs, wherein attention needs to be directed toward both pulmonary vessels and the lung [37, 38].

Third, RAPID-PH-CT was beneficial in assessing pulmonary abnormalities and assigning their relative priority compared to other findings. Specifically, compared to CRc, readers using RAPID-PH-CT effectively identified most group III PH cases (12/16 for RR4 and 16/16 for RR3). On the other hand, readers without RAPID-PH-CT properly allocated only around half of the cases (8/16 for RR1 and 7/16 for RR2). In clinical practice, distinguishing between group I and group III PH can be challenging [39], and physicians should reckon with cases displaying mixed PH phenotypes, i.e., cases that share characteristics of various PH groups rather than identify in a unique one [40]. Therefore, even if the final classification of PH derived from a comprehensive multidisciplinary evaluation may not accomplish the CT-derived hypothesis, the radiological assessment of parenchymal abnormalities remains critical. Indeed, CT-detected signs of lung fibrosis and emphysema in PH patients suggest chronic hypoxia [41] and are associated with significantly poorer survival, particularly when coupled with reduced DLCO [42].

The inter-reader agreement between RRs and CRc regarding PH etiology was substantial when using RAPID-CT-PH and only fair-to-moderate without employing it. We built RAPID-CT-PH as a comprehensive collection of complex CECT semiotics to organize imaging findings according to the thoracic anatomical districts, PH groups, and subgroup etiologies. It is worth underlining that CECT cannot claim to identify the PH etiology, and a multidisciplinary approach integrating imaging, functional, and laboratory tests is necessary to approach such a target. However, utilizing RAPID-CT-PH to browse the various conditions associated with each PH group may assist non-dedicated radiologists in avoiding the omission of subgroup-specific CT findings that can be subtle or not obvious, e.g., within group I PH, lung mosaic pattern or centrilobular ground-glass nodules in the “idiopathic form” [10], and macroscopic left-to-right cardiac shunts in the “congenital heart disease form” [1].

Using RAPID-CT-PH positively impacted the reporting completeness of chest CECT. Readers in the “control” group frequently omitted specific information that may be required for management decisions as part of the PH multidisciplinary evaluation. For instance, omitting to explicitly report the absence of notable lung abnormalities, e.g., fibrosis or emphysema, may require the referring clinician to subsequently contact the radiologist, ask for a second opinion, or even repeat the diagnostics, thus resulting in time and energy loss and, ultimately, potential management delays.

RAPID-CT-PH aided RRs in shortening the CECT reporting time. This result aligns with previous studies on diverse chest CT scenarios, e.g., COVID-19 pneumonia [43] and connective tissue diseases [44], showing the positive impact of digital supporting tools on the readers’ interpretation time. Of note, when using RAPID-CT-PH, RRs had a mean reporting time for chest CECT of 15 min, comparable (if not shorter) to large-scale radiology information system-derived radiologists’ mean reporting times, ranging from 17 to 19 min [45].

In the rising imaging-applied artificial intelligence (AI) era, emphasizing human intelligence (HI) may seem questionable. Contemporary RRs face increasing information demand on each imaging scenario, starting from the basics, such as terminology, definitions, and classifications. A direct comparison between HI and AI focusing solely on such “factual knowledge” components [46] would likely demonstrate the superiority of AI tools over humans. It has been advocated that prioritizing the reasoning processes involved in clinical-radiological integration, thereby operating at a higher level of knowledge, is essential to enhance RR education [47]. In response to this call, we set up this study involving RRs in a specific subspecialty topic, PH, to provide them with a challenge that could prototype a wide range of imaging scenarios. The present study aligns with endeavors toward implementing competency-based medical education (CBME) initiatives within radiology residency programs [48].

Some study limitations warrant mention. First, as the readers were aware that all cases were PH-confirmed, we did not evaluate the impact of RAPID-CT-PH in identifying PH in suspected cases. Therefore, no control cases without PH have been included in the study cohort. However, we intended RAPID-CT-PH to help classify rather than identify PH at CECT. We also believe that RAPID-CT-PH could provide a reliable basis for integrating chest CT-dedicated AI-driven tools. Further research is needed to explore the potential benefits of a human–machine partnership approach [49, 50], combining AI and HI to enhance imaging-derived PH phenotyping. Second, 7/60 CECTs were acquired via VPCT protocol, presumably less accurate in detecting the vascular signs of group IV PH (CTEPH). Due to the potential of VPCT protocol for effectively revealing signs related to the other PH groups [24], the influence of such a protocol heterogeneity on the reliability of PH classification is likely limited. Third, not all patients had an RHC-based diagnosis of PH; instead, a high probability of PH at echocardiography was used in 21 out of 60 cases. Although this may appear as a suboptimal patient selection, according to the 2022 ESC/ERS Guidelines on PH, RHC can be omitted in certain conditions, e.g., in patients with a high likelihood of left heart disease as the leading cause of PH [1]. We, therefore, believe this compromise does not undermine our results regarding RAPID-CT-PH. Last, this study was conducted at a single center with a small sample size, which may have led to an unbalanced distribution of cases among the groups, thus potentially hampering reproducibility. Indeed, we reported only one group V PH case related to sarcoidosis. Nonetheless, it is worth mentioning that most cases were attributed to left heart disease (group II PH), followed by lung disease (group III PH), which aligns with the frequencies reported in previous studies [2].

In conclusion, we observed substantial agreement on chest CECT-based PH classification between inexperienced readers using RAPID-CT-PH and expert radiologists. RAPID-CT-PH improved report completeness and reduced reporting time for chest CECT. Our results suggest that a quick hands-on tool for classifying PH on chest CECT can enable inexperienced radiologists to play a valuable role within the PH multidisciplinary team, thus impacting clinical decision-making. RAPID-CT-PH should be used early and systematically during the residency.

## Supplementary Information

Below is the link to the electronic supplementary material.

Supplementary file1 (PDF 37 KB)
