# Supplementary material for: Impact of an expert-derived, quick hands-on tool on classifying pulmonary hypertension in chest computed tomography: a study on inexperienced readers using RAPID-CT-PH
Source: Radiol Med. 2024 Jul 24;129(9):1313–28. doi: 10.1007/s11547-024-01852-5 (PMC11379776; doi:10.1007/s11547-024-01852-5)
Supplement: Supplementary file 1 — Supplementary file1 (PDF 37 KB) [file 11547_2024_1852_MOESM1_ESM.pdf]

La Radiologia Medica _ Impact of an expert-derived, quick hands-on tool on classifying pulmonary hypertension in chest computed tomography: a study on inexperienced readers using RAPID-CT-PH.

L Cereser, G Zussino, C Cicciò, A Tullio, C Montanaro, V Collini, M Driussi, E Di Poi, V Patruno, C Zuiani, R Girometti

Corresponding Author: Prof. Lorenzo Cereser, MD. e-mail: lcereser@sirm.org. Institute of Radiology, Department of Medicine, University of Udine, University Hospital S. Maria della Misericordia – Azienda Sanitaria-Universitaria Friuli Centrale (ASUFC), p.le S. Maria della Misericordia, 15 – 33100 Udine, Italy

Supplementary material. Chest contrast-enhanced CT examination technique

CT parameters	Venous-phase chest CT (VPCT)	CT pulmonary angiography (CTPA)
Tube potential	120 kV	100 kV
Tube current modulation range	70-400 mA	100-625 mA
Gantry revolution time	0.6 sec	0.5 sec
Detector configuration	64 x 0.625 mm	64 x 0.625 mm
Reconstruction section thickness and interval	1.25 mm	1.25 mm
